# Association between loneliness, self-esteem and outcome of life satisfaction in Norwegian adolescents aged 15–21

**DOI:** 10.1177/14034948221081287

**Published:** 2022-03-31

**Authors:** Unni K. Moksnes, Hanne N. Bjørnsen, Regine Ringdal, Mary-Elizabeth B. Eilertsen, Geir Arild Espnes

**Affiliations:** 1 Department of Public Health and Nursing, NTNU Centre for Health Promotion Research, Norwegian University of Science and Technology, Norway; 2 Trondheim Municipality, Norway

**Keywords:** Loneliness, well-being, self-esteem, youth

## Abstract

**Aims:**

Adolescence is an important developmental stage for understanding the role of perceived loneliness and self-esteem on life satisfaction. This study investigated the association between loneliness, self-esteem and the outcome of life satisfaction, as well as potential interaction effects in association with life satisfaction, in a sample of Norwegian adolescents.

**Methods:**

The study was based on a cross-sectional sample of 1816 adolescents aged 15–21 years. Data were collected in September 2016. The participants reported scores on the five-item Satisfaction with Life Scale, the 10-item Rosenberg Self-Esteem Scale and one item assessing loneliness. Control variables included sex, age, perceived family economy, parents’ education, place of birth and perceived bullying. The data were analysed with descriptive and multiple linear regression analysis.

**Results:**

A significant negative and moderately strong association was found between loneliness and life satisfaction, where the association was stronger for girls than for boys. Self-esteem showed a significant positive and strong association with life satisfaction; however, no significant interaction effect was found.

**Conclusions:**

**The findings show the significant role of both loneliness and self-esteem in association with adolescents’ perception of life satisfaction. The findings support promoting self-esteem, belongingness and social integration in all daily life contexts for adolescents to support their life satisfaction.**

## Background

Subjective well-being (SWB) is an important construct for understanding an individual’s overall functioning and quality of life. Research suggests a three-dimensional structure of SWB consisting of high positive affect, low negative affect and life satisfaction. Life satisfaction represents the cognitive component of SWB and refers to an individual’s cognitive appraisal of her or his overall quality of life according to a set of self-defined criteria [1]. Adolescence is a distinct period related to variations in life satisfaction because of the multitude of biological, psychological, social and cognitive changes occurring during this life stage [2,3]. Consistent with findings in the adult population, most studies show that a large proportion of children and adolescents report their life satisfaction to be in the positive range [1,3]. The relationship between sociodemographic factors – that is, age, sex, socioeconomic status (SES) – and life satisfaction is reported to be weak and research has shown that these variables contribute modestly to the prediction of adolescent life satisfaction. However, noted differences indicate that life satisfaction declines slightly with the onset and progression of adolescence and that boys tend to score higher on life satisfaction than girls [1,3]. Adolescents with higher SES report higher life satisfaction than those with lower SES [1,3].

Life satisfaction in adolescents is related to a broad spectrum of psychological, behavioural, interpersonal and intrapersonal factors [1]. One factor with potential impacts on adolescents’ life satisfaction is loneliness [4], which can be defined as ‘the unpleasant experience that occurs when a person’s network of social relations is deficient in some important way, either quantitatively or qualitatively’ [5]. Loneliness affects people of all ages and can vary in frequency, intensity and duration [4,6]. Recent Norwegian national reports show that 11% of the adult population (particularly those younger than 35 years and those living alone) report being very lonely [7], which corresponds with reports of loneliness in the adolescent population [2]. The evidence is inconsistent regarding whether or not there is a clear increasing trend in loneliness; however, the reported level of loneliness appears to have increased among students [8] and adolescents [9] during the last decade. Several consequences of the COVID-19 pandemic, including social distancing, actualise the importance of understanding loneliness for perception of life satisfaction among students and adolescents, a group for which there are concerning reports of increased loneliness, distress and reduced well-being [10–12].

Loneliness is considered an important public health concern due to its strong associations with various negative health outcomes and morbidity [13] as well as increased risk of mortality [14]. Studies conducted with adolescents and young adults report clear associations between loneliness and mental health problems, including symptoms of depression and anxiety [15,16]. Comparatively few studies have investigated how loneliness may impact adolescents’ perceptions of positive outcomes such as life satisfaction, although a number of studies have found support for negative associations between loneliness, mental well-being and quality of life [16–21]. Studies investigating how potential personal protective factors such as self-esteem may interact with the association between loneliness and life satisfaction are lacking.

Theories on coping indicate that personal characteristics such as self-esteem affect the association between adverse life experiences and life satisfaction. Self-esteem is defined as an individual’s set of thoughts and feelings about his or her own worth and importance [22]. The significance of self-esteem is underscored by decades of theory development and research supporting its link with, for example, mental well-being and life satisfaction [1,23,24]. Conversely, low self-esteem has been found to be related to symptoms of depression, anxiety [23,25,26] and loneliness [27,28]. In the face of challenging life circumstances, individuals with higher self-esteem are assumed to exhibit more positive coping and adjustment strategies, which may further protect the individual’s health and well-being [27,29]. However, previous research has shown varying and limited support for the moderating role of self-esteem in relation to negative life events [30,31].

Life satisfaction has been studied extensively in the adult population. Research on life satisfaction among adolescents has received increasing attention during recent decades [1]. Loneliness and quality of life are emphasised as important public health concerns among adolescents that call for more thorough investigation [19]. From the authors’ perspectives, there is a lack of studies investigating self-esteem as a potential moderator in the association between loneliness and the outcome of life satisfaction. To support adolescents’ positive development and healthy functioning, investigating the role of both risk and protective factors in association with adolescents’ perceptions of life satisfaction is pertinent. Investigating the roles of loneliness and self-esteem for adolescents’ perceptions of life satisfaction is particularly worthy of attention due to the significant physical, social, and emotional changes and transitions that occur during this stage of life [6].

Based on the empirical findings presented above, the aim of this study is twofold and include investigating: (1) the association between loneliness, self-esteem and the outcome of life satisfaction, controlled for sociodemographic factors; and (2) the interaction effects of sex by loneliness, sex by self-esteem, as well as loneliness by self-esteem in association with the outcome of life satisfaction.

## Methods

### Participants

The study is based on a cross-sectional survey of adolescents from five upper secondary schools in one of the largest cities in Norway; the student population of these schools ranges from 260 to 1087 students. The upper secondary schools offer a variety of vocational and general study tracks and, in general, are relatively similar in terms of sociodemographic characteristics. The questionnaire was administered to 2145 of a total of 3281 students in September 2016. In total, 2087 questionnaires were returned and 58 were left blank, resulting in a response rate of 97.3%. The exclusion criteria were students who responded only to background information (*n* = 11), were younger than 16 years and lacked written consent from parents (*n* = 169) or were older than 21 years (*n* = 91). The net sample size was 1816, where 934 participants were girls (51.4%), 871 (48.0%) were boys and 11 (0.6%) did not report sex (Table I). The mean age of the total sample was 17.02 years (SD 1.04); for boys, it was 17.00 years (SD 1.14) and for girls 17.03 years (SD 1.07).

**Table I.** Descriptive characteristics of the sample.

Variable	Total number (%)	No. of boys (valid %)	No. of girls (valid %)
*Sex*	1805 (99.4)	871 (48.3)	934 (51.7)
Missing data	11 (0.6)		
*Place of birth*			
Born in Norway	1677 (92.3)	808 (45.1)	862 (48.1)
Born in another country	123 (6.8)	57 (3.2)	66 (3.6)
Missing data	16 (0.9)		
*Family economy*			
Bad economy all the time	34 (1.9)	12 (0.7)	22 (1.2)
More or less bad economy	76 (4.2)	24 (1.4)	52 (2.9)
Neither bad or good economy	406 (22.4)	194 (10.9)	211 (11.9)
More or less good economy	580 (31.9)	292 (16.5)	285 (16.2)
Good economy all the time	683 (37.6)	334 (18.8)	346 (19.5)
Missing data	37 (2.0)		
*Loneliness*			
Never or very rarely	399 (22.0)	267 (15.1)	132 (7.5)
Rarely	463 (25.5)	253 (14.3)	207 (11.7)
Sometimes	626 (34.5)	236 (13.3)	388 (21.9)
Often	195 (10.7)	57 (3.2)	137 (7.7)
Very often	92 (5.0)	31 (1.8)	60 (3.5)
Missing data	41 (2.3)		
Parents’ education	Mother/father	Mother/father	Mother/father
Primary and lower secondary school	70 (3.9)/91 (5.0)	26 (1.5)/37 (2.1)	44 (2.5)/54 (3.1)
Upper secondary school	302 (16.6)/355 (19.5)	148 (8.5)/183 (10.6)	154 (8.8)/170 (9.9)
University (⩽4 years)	452 (24.9)/308 (17.0)	209 (11.9)/138 (8.0)	241 (13.8)/169 (9.8)
University (>4 years)	455 (25.1)/453 (24.9)	212 (12.1)/211 12.3)	240 (13.7)/239 (13.9)
Unknown	478 (26.3)/522 (28.7)	246 (14.1)/259 (15.0)	230 (13.1)/262 (15.3)
Missing data	59 (3.2)/ 87 (4.9)		
Total	1816 (100)		

### Procedures

The study was approved by the Regional Committee for Medical Research Ethics Mid-Norway (REK 2014/1996) and conducted in September 2016. Adolescents were informed that participation was voluntary and anonymous. Information about the study was provided by written hard copy letters to all adolescents and a video prepared by the research group, which was made available on the five schools’ e-learning platforms. The information letter was also read aloud by teachers prior to distributing the questionnaires. Students ⩾16 years gave consent to participate by answering the questionnaire, whereas written parental consent was required for students <16 years. Adolescents who chose not to participate could do other types of schoolwork. Teachers administered the questionnaires during a 45-minute classroom session.

### Measures

Life satisfaction was assessed using the Satisfaction with Life Scale (SWLS) [32]. The five-item instrument is rated on a seven-point Likert scale ranging from (1) strongly disagree to (7) strongly agree, where a higher sum score (5–35) indicates higher life satisfaction. Scores of 5–9 indicate being extremely dissatisfied with life, 10–14 dissatisfied and 15–19 slightly dissatisfied. A score of 20 represents a neutral point on the scale. Scores of 21–25 indicate being slightly satisfied, 26–30 satisfied and 31–35 extremely satisfied [32]. Examples of items include ‘In most ways my life is close to my ideal’ and ‘The conditions of my life are excellent’. The SWLS has been used extensively and found to be appropriate for assessing life satisfaction in both adults and adolescents [33]. The internal consistency of the SWLS in this study showed a Cronbach’s α of .89.

Self-esteem was measured using the Rosenberg Self-Esteem Scale (RSE) [34], a 10-item questionnaire measuring global self-esteem. The items are rated on a four-point Likert scale ranging from (1) strongly disagree to (4) strongly agree, where a higher sum score (range 10–40) indicates a higher level of global self-esteem. No cut-off point has been established for the scale in reference to low and high self-esteem. The RSE is a reliable and valid measure for global self-esteem, including the adolescent population [23]. Cronbach’s α for the present study was .90.

Loneliness was measured using a one-item variable worded as ‘Do you ever feel lonely?’ The options were (1) never or very rarely, (2) rarely, (3) sometimes, (4) often and (5) very often. This item has been used in prior studies of loneliness [35].

Bullying was assessed by three items that asked adolescents how often they experienced the following: ‘Your peers accuse you of things you have not done or cannot help’; ‘Your peers show that they do not like you, e.g. by teasing, whispering or making fun of you’; and ‘One or more peers hit you or hurt you in other ways’. The response values included (1) never, (2) occasionally, (3) at least once a month, (4) at least once a week and (5) almost every day. The item responses were expressed as a sum score for which higher scores indicate a higher exposure to bullying (range 3–15). Cronbach’s α for the present study was .70. These items are included in the national Norway Survey of Living Conditions 2012–2013 [36].

Sociodemographic variables included sex, age, SES and place of birth. SES was measured in terms of parents’ education and adolescents’ perceptions of their family’s economic situation, variables that were included in a previous study [16]. Place of birth was assessed by one item: ‘In what country were you born?’ The possible responses were (1) Norway and (2) in another country. Parental education was assessed separately with the item ‘What is the highest level of education that your parents have attained?’ The responses included (1) primary and lower secondary school, (2) upper secondary school, (3) university ⩽4 years, (4) university >4 years and (5) I don’t know. In the multivariate analysis, parental education was used as an index by computing a sum score range of 2–10. Adolescents’ perceptions of their family’s economic situation was assessed with the item ‘How has your family’s economic situation been during the last two years?’ Response items were (1) we have had a bad economic situation the whole time, (2) we have had a more or less bad economic situation, (3) we have had neither a bad nor good economic situation, (4) we have had a more or less good economic situation and (5) we have had a good economic situation the whole time.

### Statistical analyses

Statistical analyses were conducted using IBM SPSS for Windows v24.0 (IBM Corp., Armonk, NY, USA). An independent samples *t*-test was used to investigate sex mean differences on the scales; effect sizes (Cohen’s *d*) were calculated following guidelines for small (.20), medium (.50) and large (>.80) effect sizes. Bivariate associations were tested with Pearson’s correlation. Multiple linear regression analysis was used to investigate associations between loneliness, self-esteem and the outcome of life satisfaction, controlled for sex, age, SES, place of birth and bullying. Interaction effects were tested including sex × loneliness, sex × self-esteem and self-esteem × loneliness. The proportions of missing values for the variables of loneliness, self-esteem and life satisfaction varied in the range 2.3–8.5%. In the survey, the value ‘I don’t know’ was included for the variable of parents’ education to ensure valid responses from the participants. In the multivariate analysis, this value was excluded to have a continuous variable. When constructing scale sum scores, cases with missing responses in the proportion of ⩽20% for each scale were included.

The variables of life satisfaction, self-esteem, loneliness, bullying, parents’ education and family economy were slightly skewed as indicated by the histogram and normal *q–q* plot; however, no serious violation of normality was found. Model assumptions for linear regression analysis were tested, with no indication of multicollinearity (VIF 1.018–3.037; correlations <0.80). The assumptions of linearity, multivariate normality and independent residuals were also met by inspection of the normal *P–P* plot, scatter plot and Durbin–Watson test close to 2. Multivariate linear regression analysis was conducted with a listwise deletion and *p* ⩽0.05 was considered statistically significant.

## Results

### Descriptive statistics

Table I presents the descriptive characteristics of the sample. Regarding place of birth, the majority of adolescents were born in Norway and 6.9% were born in another country. A majority also reported having a good family economy and parents with higher education at university level. Regarding loneliness, 15.7% reported being lonely ‘often’ or ‘very often’, 34.5% reported being lonely ‘sometimes’ and 47.5% reported being lonely ‘rarely’ or ‘very rarely/never’.

### Mean scores and correlations of study variables

Table II presents the mean scores and bivariate correlations for the study variables. When looking at life satisfaction, the mean score was at the neutral point of the scale (⩾20). Further, 7.2% of the sample had very high scores (⩾31) and only 5% had very low scores on life satisfaction (⩽9) (not shown). The mean scores on self-esteem were also at the positive end of the scale. Results from the independent samples *t*-test showed that boys scored significantly higher than girls on self-esteem, life satisfaction and family economy, whereas girls scored significantly higher on loneliness. Sex differences presented small to medium effect sizes. The bivariate correlations of the study variables showed significant and strong correlations of life satisfaction with both self-esteem and loneliness. Regarding the sociodemographic variables, family economy showed significant positive correlations with life satisfaction, whereas bullying showed a significant negative correlation with life satisfaction.

**Table II.** Mean scores and correlations between study variables.

		Mean (SD)	Range	*t*-value	*P*-value	Cohen’s *d*	1	2	3	4	5	6	7
1. Satisfaction with life	Total	21.56 (7.02)	5–35					.66**	−.52**	−.23**	.06*	.27**	−.10**
	Boys	22.89 (6.91)	5–35	−7.26	.000	.36							
	Girls	20.41 (6.90)	5–35										
2. Self-esteem	Total	28.16 (6.40)	10–40						−.55**	−.29**	.08**	.20**	−.01
	Boys	30.29 (6.18)	10–40	−13.13	.000	.65							
	Girls	26.32 (6.00)	10–40										
3. Loneliness	Total	2.77 (1.07)	1–5							.28**	−.01	−.22**	.06*
	Boys	2.20 (1.07)	1–5	10.95	.000	.53							
	Girls	2.77 (1.07)	1–5										
4. Bullying	Total	4.31 (1.77)	3–15								.03	−.13**	−.05*
	Boys	4.36 (1.93)	3–15	−.987	.324	.05							
	Girls	4.27 (1.61)	3−15										
5. Parents’ education	Total	5.94 (1.68)	2−10									.30**	−.08**
	Boys	5.94 (1.68)	2–10	.18	.857	.01							
	Girls	5.95 (1.69)	2–10										
6. Family economy	Total	4.01 (.98)	1–5										−.08**
	Boys	4.07 (.92)	1–5	−2.23	.026	.11							
	Girls	3.96 (1.03)	1–5										
7. Age	Total	17.02 (1.04)	15–21	.509	.610	0							
	Boys	17.00 (1.14)	15–21										
	Girls	17.03 (1.07)	15–21										

Cohen’s *d*-effect sizes: small (0.20), medium (0.50) and large (>0.80).

* *p* ⩽ .05; **p ⩽ .01.

### Multiple linear regression analysis for the associations between sociodemographic variables, loneliness, self-esteem and life satisfaction

Table III presents the results from the multiple linear regression analysis for associations between loneliness, self-esteem and the outcome of life satisfaction, adjusted for sex, age, SES, bullying and place of birth. Both unadjusted and adjusted results showed that perceived loneliness was significantly negatively associated with life satisfaction, whereas self-esteem showed a significantly strong, positive association with life satisfaction. Significant interaction effects were evident between loneliness by sex in association with life satisfaction, where the association was significantly stronger for girls (*B* = −1.70; CI −2.11 to −1.30) than for boys (*B* = −.95; CI −1.45 to −.44); the other interaction effects were not significant in association with life satisfaction. Overall, the regression model explained 49% of the variance in life satisfaction.

**Table III.** Multiple linear regression analysis for the association between loneliness, self-esteem and outcome of satisfaction with life, adjusted for sex, age, socioeconomic status, place of birth and bullying.

	Satisfaction with life								
	Unadjusted			Adjusted					
	*B*	*β*	95% CI	*B* ^a^	*β*	95% CI	*B* ^b^	*β*	95% CI
Loneliness	−3.27	−.52***	−3.54 to −3.01	−1.74	−.28***	−2.16 to −1.31			
Self-esteem	.72	.66***	.68–.76	.55	.51***	.47–.63			
Sex × loneliness							.83	.08*	.19–1.47
Sex × self-esteem							.04	.02	−.08–.15
Self-esteem × loneliness							.01	.02	−.03–.06

*** Estimates significant at *p* ⩽ .001 *estimates are significant p ⩽ .05.

Unadjusted analyses present bivariate estimates for loneliness and self-esteem.

a Adjusted for sex, age, parents’ education, family economy, place of birth and bullying.

b Adjusted for sex, age, parents’ education, family economy, place of birth, bullying, self-esteem and loneliness.

## Discussion

This paper furthers our understanding of the association between loneliness, self-esteem and the outcome of life satisfaction in adolescents. Three findings are highlighted in this study: (a) the modest role of sociodemographic factors in association with life satisfaction, although significant differences were found; (b) the significantly negative and moderately strong association between loneliness and life satisfaction, especially for girls; and (c) the significantly strong positive association between self-esteem and life satisfaction, where a significant interaction of sex by loneliness was found.

Similar to previous findings [1], the descriptive results showed that the level of life satisfaction in this study was in the positive range, with average mean scores (mean score ⩾20), indicating that the majority of adolescents report being generally satisfied with their lives. These findings are in line with previous studies on adolescents showing mean scores between 23.0 and 25.0 [32]. The initial results from the *t*-test and bivariate correlations showed that, of the sociodemographic factors, family economy was the factor which associated moderately strongly and significantly with life satisfaction, followed by bullying. Boys scored higher on life satisfaction than girls. The factors parents’ education and age were weakly associated with life satisfaction. The findings correspond with previous studies showing that sociodemographic factors (sex, age and SES) contribute modestly to adolescents’ reported life satisfaction, although variations are normative during adolescence [1,3,12].

With reference to loneliness, most of the adolescents reported that they ‘rarely’ or ‘never’ experienced being lonely, whereas 11% reported being lonely ‘often’ and 5% ‘very often’. These findings are in line with national reports of loneliness among adolescents [2]; however, the report of loneliness increased during the period with social restrictions related to the COVID-19 pandemic [10]. The significant sex differences found in loneliness in the bivariate results showing higher levels in girls are in line with previous studies in adolescents [4,8,10] and adults [37], although sex does not seem to predict loneliness over the life course [15,38].

The negative and moderately strong association found between loneliness and life satisfaction in the multivariate results is also in accordance with previous research [19,20]. As social beings, humans have a basic ‘need to belong’ and the desire for meaningful relationships is important for the perception of quality of life over the life course [4]. By contrast, loneliness is a negative or distressing emotion that accompanies the perception that one’s social needs are not being adequately met. Social acceptance and belonging in relation to one’s peer group is particularly important during adolescence [10,39]. Consequently, being lonely may include not only the feeling of being alienated from peers, but also of failing the critical task of being socially connected. Loneliness is heterogeneous during childhood and adolescence and may follow different developmental trajectories [40]. When the experience of loneliness becomes chronic, it may negatively affect the individual’s mental health and life satisfaction [17,18]. Interestingly, the association between loneliness and life satisfaction was stronger for girls than for boys, as indicated by the significant interaction effect in the regression analysis. One explanation for the stronger association found for girls could be related to differences in gender roles and friendship interactions, which may lead to different thresholds for reporting loneliness. In a broader social context, girls may be more sensitive to interpersonal aspects of the social environment and more emotionally expressive than boys [16,38]. These aspects may result in different expectations regarding personal roles and relationships with friends, which may lead to girls perceiving greater intrinsic loneliness than boys when these expectations are not met. Consequently, the experience of loneliness may potentially affect girls’ perceptions of life satisfaction more negatively than for boys. However, we should also be aware of potential bias in relation to gender roles and self-reporting of these aspects that may influence how boys and girls answer these questions.

When it comes to self-esteem, the sample mean scores were in the positive range, with significantly higher scores in boys than girls, which is in accordance with previous studies [24,25]. The significant and strong association found between self-esteem and life satisfaction in the regression analysis aligns with previous studies showing self-esteem to be an important factor for maintaining psychological health, well-being and positive functioning during adolescence [1,23,24]. Self-esteem includes the evaluative and affective dimensions of the self-concept and is likely to vary during adolescence in relation to personal and social changes and transitions [22]. Individuals with higher self-esteem are assumed to show better adjustment and positive coping in relation to challenges and adversities [22]. They may also seek and receive more social support, which may facilitate positive coping behaviours and overall adjustment. By contrast, individuals with low self-esteem may be perceived as having less confidence and a lower ability to identify resources for intended purposes, which may affect life satisfaction negatively.

The present findings support promoting adolescents’ personal and social–emotional resources; such support is crucial for the development of self-esteem and life satisfaction in adolescents [1,24]. With reference to loneliness, it is important to promote social support and connectedness in different settings in adolescents’ daily lives, including school, leisure time activities and the local community.

### Strengths and limitations

The particular strengths of this study are the use of validated instruments, the large sample size and the high response rate. However, the results should be interpreted without any reference to causal conclusions based on the cross-sectional study design. A small proportion of the sample (6.8%) was not born in Norway; this is significantly less than the general population in Norway [41]. Thus, the results may not be representative of Norway’s adolescent population overall. Further, a single item was used to measure loneliness, which assessed its frequency. The phenomenon of loneliness is complex and therefore should be assessed by an instrument that includes variations in intensity, circumstances and time, as well as both direct and indirect questions about loneliness [42]. This study was based on self-reports from adolescents. Although self-reporting is a well-used method for assessing subjective health in adolescents, it may also present potential challenges with reference to self-report bias. These aspects refer to social desirability, over- and under-reporting due to potential social stigma and gender role bias in regard to reporting life satisfaction, loneliness, self-esteem and SES. However, the large sample size is thought to be a strength of the study protecting the results from the influences of potential bias related to self-reporting.

## Conclusions

The study supports the significant negative relationship between loneliness and life satisfaction, where stronger associations were found for girls. The study also supports the theoretical and empirical understanding of self-esteem as a positive personal factor associated with life satisfaction controlled for loneliness and sociodemographic factors. Although the present study does not allow for causal conclusions, the findings support the relevance of both loneliness and self-esteem for adolescents’ perceptions of life satisfaction.
